# Recent Advances in C_5_ and C_6_ Sugar Alcohol Synthesis by Hydrogenation of Monosaccharides and Cellulose Hydrolytic Hydrogenation over Non-Noble Metal Catalysts

**DOI:** 10.3390/molecules27041353

**Published:** 2022-02-17

**Authors:** Elena Redina, Olga Tkachenko, Tapio Salmi

**Affiliations:** 1 N.D. Zelinsky Institute of Organic Chemistry, Russian Academy of Sciences, 47 Leninsky Prospect, 119991 Moscow, Russia; ot@ioc.ac.ru; 2 Johan Gadolin Process Chemistry Centre, Abo Akademi University, FI-20500 Turku, Finland; tapio.salmi@abo.fi

**Keywords:** sugar alcohol, cellulose, glucose, sorbitol, xylitol, mannitol, hydrogenation, hydrolytic hydrogenation, nickel, copper, non-noble metals, catalysts

## Abstract

A new reality of the 21st century is the transition to a new type of economy and energy concepts characterized by the replacement of existing petrochemical routes to a bio-based circular economy. The needs for new strategies in obtaining basic products from bio-based resources with minimum CO_2_ traces has become mandatory. In this review, recent trends in the conversion of biomass-derived molecules, such as simple monomeric sugars and cellulose, to industrially important C_5_ and C_6_ sugar alcohols on heterogeneous catalysts based on non-noble metals are discussed focusing on the influence of catalyst structures and reaction conditions used on the substrate conversion and product selectivity. The challenges and prominent ideas are suggested for the further development of catalytic hydrogenation of naturally abundant carbohydrates to value-added chemicals on non-noble metal catalysts.

## 1. Introduction

Over the past 10 years, green chemistry has become not just a fashionable area of research, but a new reality. The transition from the traditional fossil-based economy and petrochemical technologies to a new era of bio-based circular economy is encouraged by the reduction in an over-dependency on fossil resources and minimization of the impact on the environment. The new type of bio-based chemical technology implies the replacement of petroleum-derived chemicals by bioavailable platform substances. In the period 2004–2017, the US National Renewable Energy Laboratory (NREL) and EU-funded project RoadToBio have defined top value-added chemicals from biomass. Among the top 12 platform molecules, polyalcohols such as sorbitol, xylitol, and arabinitol were listed [1]. These alcohols are the hydrogenation products of corresponding C_6_ and C_5_ sugars. Thus, for instance, sorbitol is a product of glucose hydrogenation or cellulose hydrolytic hydrogenation, while xylitol and arabinitol are obtained by hydrogenation of xylose and arabinose.

**Figure 1:**
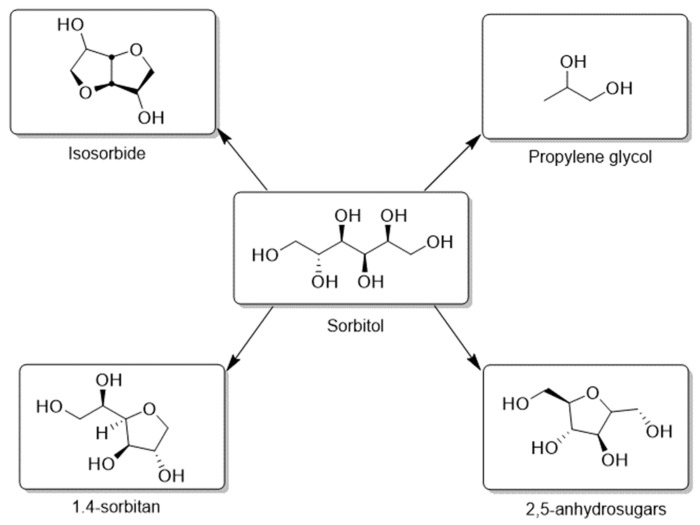
Value-added chemicals from sorbitol.

**Figure 2:**
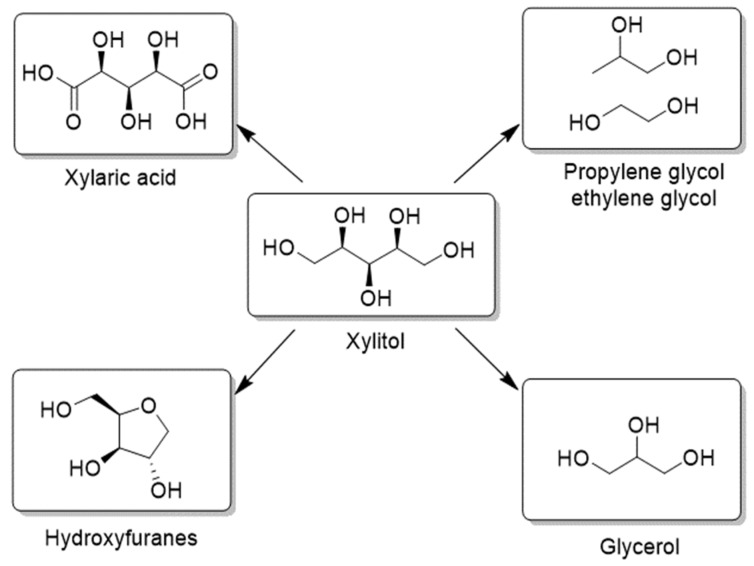
Value-added chemicals from xylitol.

C_5_ and C_6_ sugar alcohols are considered as perspective building blocks and important synthons in organic synthesis obtained from cellulose or hemicelluloses [2,3,4]. Sorbitol can be consumed not only directly as a sugar substitute or in cosmetics but also in the synthesis of tetrahydrofuran derivatives and glycols (Figure 1). Sorbitol represents a substrate for isosorbide production at low cost. Isosorbide has been shown to be a very effective monomer in copolymerization with PET in order to increase the temperature resistance of polymer products, for example, plastic bottles [5]. Xylitol and arabinitol are usually used as sweeteners instead of sugar. Xylitol has antibacterial effects against streptococci bacteria; thus, it reduces dental plaque, and it might be protective against tooth decay and actively used in toothpastes. These sugars can also be important substrates in the production of glycols [6] as well in the synthesis of hydroxyfuranes—the building blocks in pharmaceutical industry [7] (Figure 2). 

Catalytic hydrogenation of C_5_ and C_6_ sugars is usually performed over supported and unsupported noble mono- and bimetallic nanoparticles (Pt, Ru, Pd) [8,9,10]. However, the reaction requires harsh conditions (elevated temperatures and/or hydrogen pressure) even with noble-based metal catalysts. Najarnezhadmashhadi et al. showed that Ru nanoparticles (NPs) supported on aluminum foam can be effectively used in the hydrogenation of glucose, arabinose, and galactose in continuous mode at 110 ^°C^ and a hydrogen pressure of 20 atm [11]. Xylitol, arabinitol, sorbitol, mannitol, and galactitol were obtained by the hydrogenation of corresponding sugars over Ru/MWCNT at 110 °C and hydrogen pressure of 30 atm [12]. Simakova et al. also used Ru/C for the hydrogenation of arabinose and galactose and showed the structure sensitivity of this reaction. The highest activity was obtained over the catalyst with Ru NPs of mean size ca. 3 nm at 110 °C and a hydrogen pressure of 20 atm [13].

Homogeneous hydrogenation of sugars over noble-metal complexes requires harsh conditions as well [14,15]. However, homogeneous hydrogenation creates an opportunity in asymmetric hydrogenation and the formation of pure products with high stereoselectivity. Recently, Tindal et al. showed that complex [RuCl_2_(benzene)]_2_ with (S) or (R) -DTBM-SEGPHOS allowed stereoselective hydrogenation of different ketoses with quantitative yield, yet the reaction was performed at 100 °C and a hydrogen pressure of 60 atm [16]. 

The need for rather harsh conditions for sugar hydrogenation to corresponding alcohols even in the presence of catalysts based on noble metals has oriented the research towards the replacement of noble metals with non-noble ones, assuming the possibility that under such conditions, the catalysts with base metals would also be active. The transition from noble-metal-based catalysts to highly available and cost-effective catalytic systems with base metals will make the industrially important sugar hydrogenation process more popular, affordable, and economical. However, developing new catalysts based on non-noble metals with high activity, selectivity, and stability remains a big issue for scientists. In this review, we want to address the advances in C_5_ and C_6_ sugar hydrogenation and cellulose hydrolytic hydrogenation on non-noble metal catalysts in the last decade.

## 2. Hydrogenation of Glucose to Sorbitol

**Figure 3:**
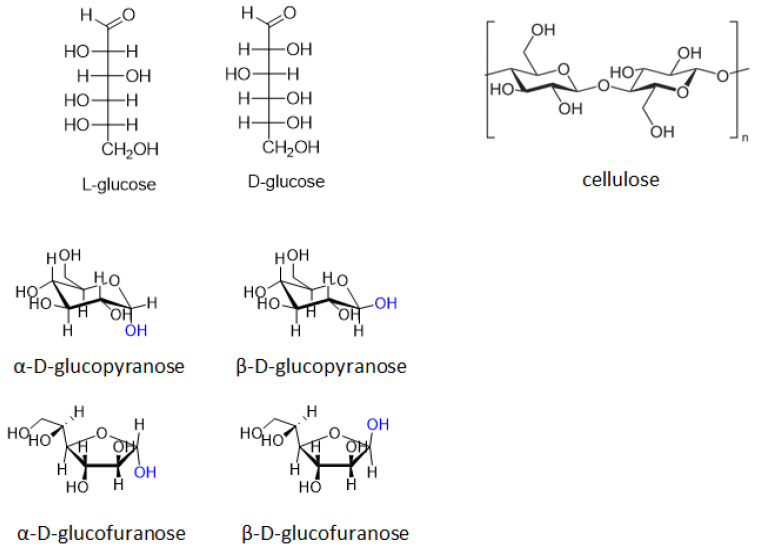
Different tautomeric and diastereomeric forms of glucose and structural fragment of cellulose.

Glucose is the most abundant monosaccharide which is formed by plants and algae during photosynthesis. Moreover, glucose is the structural link of cellulose, which consists of a linear chain of several hundreds to many thousands of β(1→4) linked D-glucose units and, thus, known to be the most common carbohydrate and natural polymer, accounting almost 50% of all carbon in biosphere (Figure 3) [17]. 

Glucose contains six carbon atoms and an aldehyde group; therefore, it is attributed to aldohexoses. It can be easily obtained by cellulose hydrolysis by enzymatic or chemical processing with the use of mineral acids or solid acid catalysts [18]. Glucose molecule can exist in an open-chain (acyclic) as well as cyclic forms, which in turn can be six-membered (α- or β-pyranose form) and five-membered (α- or β-furanose form); β-glucopyranose is the predominant form in the solution (Figure 3). 

Glucose hydrogenation to sorbitol in aqueous solution is a well-studied reaction realized on an industrial scale (700.000 tones/year) on a finely dispersed Raney Ni catalyst in batch mode [19]. Despite the excellent catalytic properties and low cost of Raney Ni, the major problem is its deactivation because of leaching of Ni species into reaction mixture; thus, an additional product purification procedure is required [20]. Therefore, researchers have tried different modifications of Raney Ni as well as the development of novel catalysts based on easily accessible non-noble metals [21,22].

Ni containing unsupported bi-metallic nanoparticles have been shown to be active catalysts for D-glucose to D-sorbitol hydrogenation (Table 1). Amorphous Ni-Co (1:1) nanoparticles prepared by mechanochemical synthesis and reduced with NaBH_4_ were able to provide almost complete glucose conversion to D-sorbitol with the yield exceeding 96% after 4 h in water at a temperature only 90 °C and 30 atm of hydrogen pressure [23]. Monometallic Ni and Co nanoparticles were less active in the reaction. The glucose conversion on Ni was 77%, while on Co NPs, the conversion reached only 26%, but the selectivity to D-sorbitol was 99% in both experiments. It is important to note that according to XPS spectra, Ni and Co in bimetallic nanoparticles were mostly in a metallic state with a small partial negative charge on Ni; boron impurities were also observed in XPS spectra. The authors claimed that the partial negative charge on Ni in Ni-Co bimetallic catalyst along with smaller sizes of Ni-Co NPs were responsible for their higher catalytic activity in glucose hydrogenation compared to monometallic Ni and Co catalysts. The calculated activation energy (*E*_a_) for Ni-Co (1:1) nanoparticles appeared to be 48.9 kJ mol^−1^ and changed within ± 0.01 kJ mol^−1^ with an increase or decrease in the Co concentration in Ni-Co NPs (Ni:Co=2:1 and Ni:Co=1:2). The authors suggested that these facts indicated that the active species for hydrogenation were the same for the samples with higher and lower Co content, possibly Ni^δ-^, where hydrogen activation could proceed [23].

**Figure 4:**
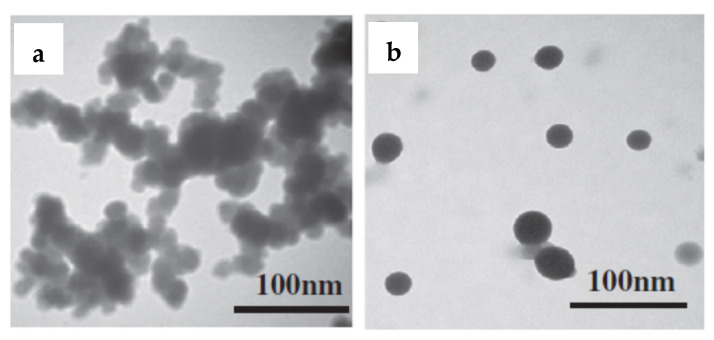
TEM images of Ni-B amorphous alloy prepared by conventional NaBH4 reduction (**a**) and by chemical reduction of nickel hydrazine complex Ni(N_2_H_4_)_2_^2+^ by BH_4_^−^ with use of ultrasound (**b**) (reprinted from Publication [24] with permission from Elsevier).

Guo et al. showed that Ni-B amorphous alloy with uniform particles (Figure 4) prepared by a reduction of nickel hydrazine complex Ni(N_2_H_4_)_2_^2+^ with BH_4_^−^ in aqueous solution (Equation (1)) induced by ultrasound provided glucose hydrogenation with the conversion of 85% after 2 h of reaction at 120 °C and P(H_2_) = 40 atm [24].

[Ni(N_2_H_4_)_2_]Cl_2_ + BH_4_^−^ + 2H_2_O → Ni^0^ + BO_2_^−^ + 2N_2_H_4_ + 2Cl^−^ + 2H_2_ (1)

According to TPR-H_2_ data and XPS analysis of Ni-B alloy, the formation of Ni^0^ due to strong back-electron donation from B together with the presence of only one adsorbing site on the catalyst surface were responsible for the rather high activity of Ni-B nano-alloy in glucose hydrogenation. Raney Ni was considerably less active, and glucose conversion reached only 38% under the same reaction conditions. 

**Figure 5:**
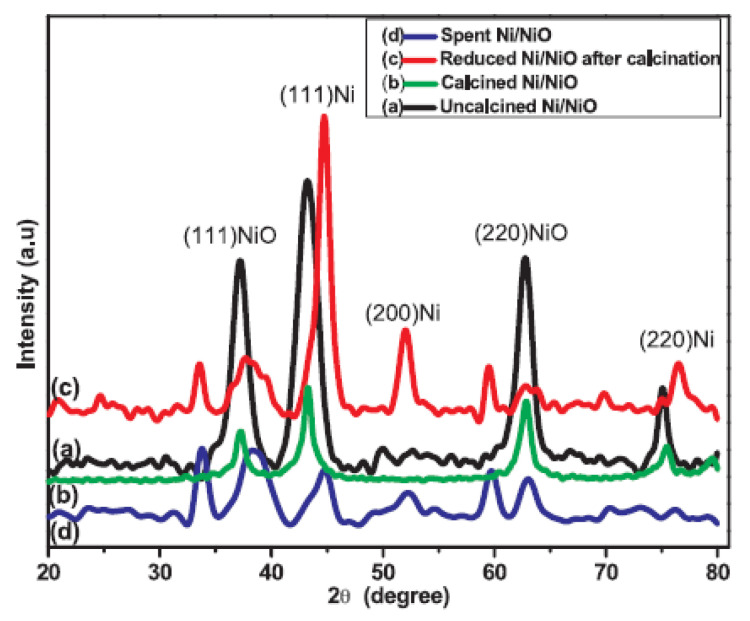
XRD patterns of (a) before calcination mesoporous Ni/NiO, (b) after calcined mesoporous Ni/NiO catalyst (c) reduced mesoporous Ni/NiO catalyst and (d) used mesoporous Ni/NiO catalyst (reprinted from Publication [25] with permission from Elsevier).

Another type of Ni unsupported NPs obtained through hydrazine hydrate Ni complexes was reported by Singh et al. [25]. Thermally stable unsupported crystalline mesoporous Ni/NiO catalyst was prepared by a nucleation-reduction method with the mixture of NaBH_4_ and hydrazine hydrate. The phase structure of the catalysts was confirmed by XRD (Figure 5). Ni/NiO NPs with a size of 11–24 nm provided D-glucose hydrogenation to D-sorbitol with conversion up to 95% and a selectivity of 88% in water at 130 °C. and hydrogen pressure of 50 atm; the other product was D-mannitol, obtained with the selectivity of 12% (Table 1). The activation energy was calculated to be 16 kJ mol^−1^, and it was four times lower than that on Raney Ni, while the stability of Ni/NiO catalyst was greatly enhanced [25]. Interestingly, Silvester et al. showed that supported 5%Ni/Al_2_O_3_ catalyst prepared by a precipitation-reduction method with hydrazine hydrate enabled D-glucose conversion of only 60% with selectivity to D-sorbitol reaching 80% at 130 °C and P (H_2_) = 30 atm after 4 h of reaction with the substrate-to-Ni ratio close to that used by Guo for Ni-B nanoalloy [24,26].

Supported catalyst 5%Cu/Al_2_O_3_ prepared by the same method was even less active under the conditions used; thus, the glucose conversion was only 45% with a selectivity to D-sorbitol of 89% [26]. A slightly higher D-glucose conversion of 80% with the D-sorbitol selectivity of 87% was observed on the reduced Cu-Ni-Al hydrotalcite precursor (Ni_1.85_Cu_1_Al_1.15_) at 130 °C and P(H_2_) = 30 atm in water after 3 h of reaction, but under a low substrate-to-metal ratio of 0.03 mol/g. The presence of Ni^0^ and Cu^0^ was also shown to be responsible for the increase in the D-glucose conversion to D-sorbitol [27]. 

**Figure 6:**
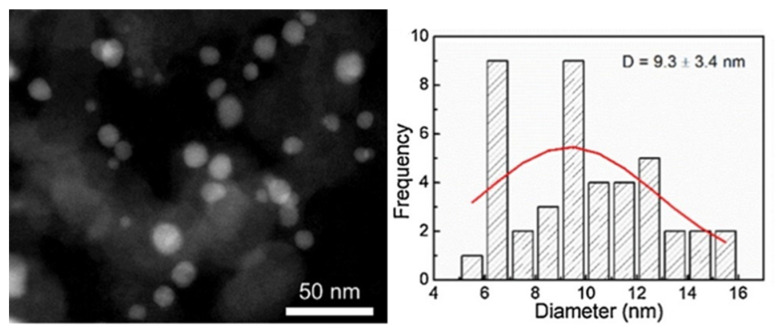
TEM image of 8%Fe-8%Ni/CB catalysts and particle size distribution (reprinted from Publication [28] with permission from Elsevier).

**Figure 7:**
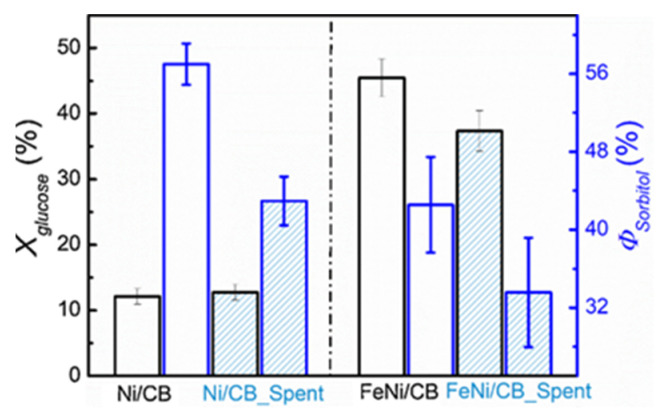
Glucose conversion (*X glucose*) and sorbitol selectivity (*Φ* *sorbitol*) over fresh and spent catalysts. Reaction conditions: 60 mg catalyst, 0.5 g glucose, 100 mL H_2_O, T = 120 °C, p (H_2_) = 30 bar, 120 min and stirring speed of 1700 rpm (reprinted from Publication [28] with permission from Elsevier).

Fu et al. prepared Fe-Ni bimetallic nanocatalyst supported on carbon black (CB) (Figure 6) [28]. The presence of Ni^0^ and Fe^0^ in alloy NPs was critical to obtain D-sorbitol from D-glucose in good yields. It was shown that on monometallic Ni/CB, the dominant pathway was glucose hydrogenation, while on Fe/CB, glucose isomerization to fructose was observed. The combination of Fe and Ni with the weight ratio 1:1 enabled to achieve high activity and selectivity to D-sorbitol whilst increasing the catalyst stability compared to that of Ni/CB. 8%Fe-8%Ni/CB catalyst provided a D-glucose conversion of 71% with a selectivity to D-sorbitol of 70% at 140 °C and H_2_ pressure of 40 atm after 6 h of reaction. It should be noted that Ni leaching to water medium and growth of particle size were responsible for the deactivation of bimetallic sample, but it was less pronounced than for Ni/CB (Figure 7) [28].

## 3. Fructose Hydrogenation

Fructose is a ketonic simple C6 sugar found in many plants, root vegetables, and honey, where it is often bonded to glucose to form the disaccharide sucrose. Mannitol obtained by fructose hydrogenation is usually used as a low-calorie sweetener, and it has also found an application as a vasodilator in hypertension treatment in mannitol hexanitrate form [37].

**Figure 8:**
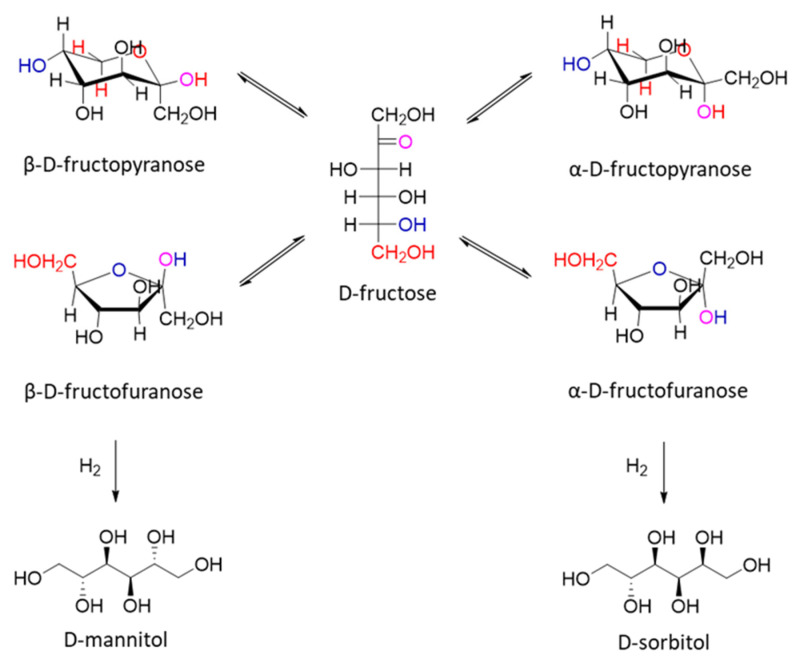
Different tautomeric forms of fructose and corresponding alcohols.

As a glucose molecule, fructose is present in solution in the form of five- and six-membered cyclic hemiketals: α– and β- D(L)-fructopyranose, α– and β-D(L)-fructofuranose. Heinen et al. suggested that the fructofuranose forms are more reactive than the fructopyranose forms in hydrogenation reactions (Figure 8) [38]. It was reported that different tautomeric forms of fructose have different adsorption strengths on the surfaces of hydrogenation catalysts, as well as hydrogenation rates [39]. According to the theory and DFT calculations, fructose hydrogenation over Cu and Ni catalysts proceeds through the hydrogenation of cyclic forms, but not by hydrogenation of the acyclic form. Moreover, in fructose hydrogenation, β-fructose molecules are converted to mannitol, while α-fructose molecules give sorbitol as the hydrogenation product. The pyranose form probably plays a minor role in the hydrogenation [29]. It should also be noted that fructose can also be isomerized to glucose in the presence of basic and acid sites [40,41]. Therefore, the main problem in the hydrogenation of fructose is the selectivity; besides mannitol, sorbitol is the second reaction product formed in a parallel reaction.

Catalysts with non-noble metals are actively used in fructose hydrogenation. On the reduced hydrotalcite-like magnetic catalyst with a composition Ni_4.63_Cu_1_Al_1.82_Fe_0.79_ and particle size of 11–14 nm, the mannitol and sorbitol yields were 57% and 43%, respectively, at complete fructose conversion under 110 °C and P(H_2_) 30 atm, but at a low substrate-to-catalyst ratio of 0.03 mol/g [30]. The author observed that with an increase in the reduction temperature of hydrotalcite catalyst, the fraction of Ni^0^ and Cu^0^ catalyst grew, which in turn led to higher fructose conversion, while the selectivity to mannitol and sorbitol remained unchanged. 

**Figure 9:**
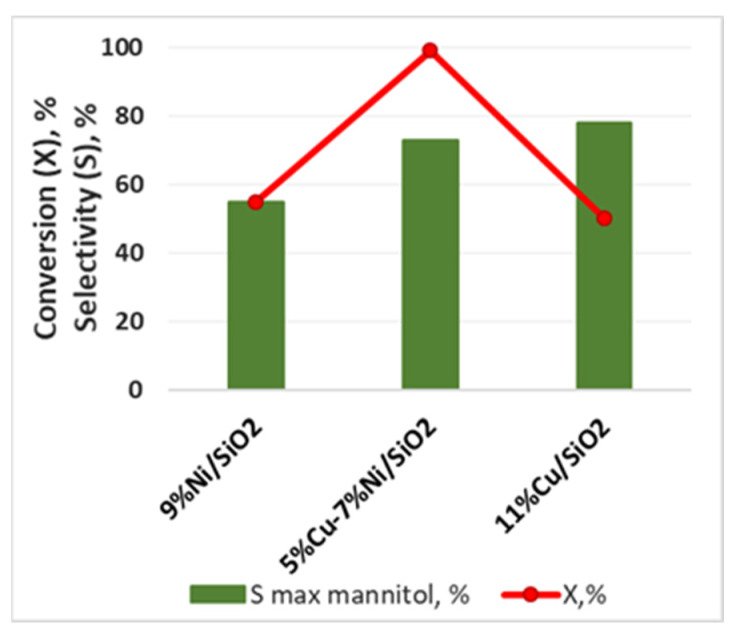
Performance of monometallic Cu/SiO_2_, Ni/SiO_2_ and bimetallic Cu-Ni/SiO_2_ catalysts in fructose hydrogenation to mannitol at 100 °C and P(H_2_) = 40 atm after 2 h of reaction in water [30].

A much better selectivity of D-fructose hydrogenation to D-mannitol was observed by Zelin et al. over supported bimetallic CuNi nanoparticles. The use of 5%Cu-7%Ni/SiO_2_ catalyst allowed the attainment of almost full D-fructose conversion with a selectivity to D-mannitol of 73% at 100 °C and P(H_2_) = 40 atm after 2 h of reaction [31]. CuNi alloy nanoparticles were more active and stable in D-fructose hydrogenation than monometallic Cu/SiO_2_ and Ni/SiO_2_ samples (Figure 9). It was proved by a number of methods (XPS, TPR-H_2_, TPD-H_2_) that the Ni surface in the Cu-Ni/SiO_2_ catalyst was modified by dilution with Cu, thus forming strong interaction between Cu^0^ and Ni^0^ phases. Despite the decline of the catalyst from cycle to cycle because of carbon residues, it can be regenerated in H_2_ at 500 °C without any change in the selectivity to D-mannitol. 

Nevertheless, a rather simple catalytic system Cu/SiO_2_ can be effectively applied in the hydrogenation of fructose. Copper catalysts favour more selective hydrogenation of fructose to mannitol than Ni-containing samples [39]. It was assumed that on small Cu^0^ NPs, fructose preferably adsorbs in β-fructofuranose form, hydrogenation of which led to mannitol with a high selectivity [42,43]. Zelin and co-workers proposed 11%Cu/SiO_2_ catalysts with highly dispersed metallic Cu NPs of 3 nm for fructose hydrogenation in a water–ethanol mixture [42]. The yield of D-mannitol of 78% at complete D-fructose conversion was achieved in 6 h at 100 °C and P(H_2_) = 40 atm. The catalyst 11%Cu/SiO_2_ with the mean Cu NPs size of 32 nm allowed only 32% D-fructose conversion and D-mannitol selectivity as low as 68%. The obtained catalysts were not stable and deactivated because of copper leaching [42]. Recently, Upare et al. prepared a series of Cu-SiO_2_ nanocomposites by a precipitation-deposition method with the use of colloidal silica and Cu(NO_3_)_2_ ·3H_2_O. This method enables the obtainment of catalysts with high Cu loadings (up to 80%) and high specific surface areas (100–400 m^2^/g) [29]. The obtained catalysts with Cu content 60-80% wt. allow full D-fructose conversion and D-mannitol selectivity up to 84% in 1-butanol solvent at 120 °C and P(H_2_) = 35 atm after 10 h of reaction. The authors showed that the hydrogenation of fructose was catalyzed by Cu metallic sites. It should be pointed out that no Cu leaching was observed during the reaction in 1-butanol, while the use of water as a solvent led to Cu leaching as well as a significant amount of the fructose degradation and/or condensation products formed as by-products on the catalyst surface. However, decline in the activity was noticed on the most active 80%Cu-SiO_2_ catalyst when its recycling, which was ascribed to the partial copper oxidation to Cu^+^ as well as fructose, strongly chemisorbed on the catalyst surface. Regeneration of the catalyst in H_2_ at 290 °C completely restored the initial activity of the catalyst, and it could show stable work for at least six cycles [29]. 

## 4. Hydrogenation of Other C_5_ and C_6_ Sugars

**Figure 10:**
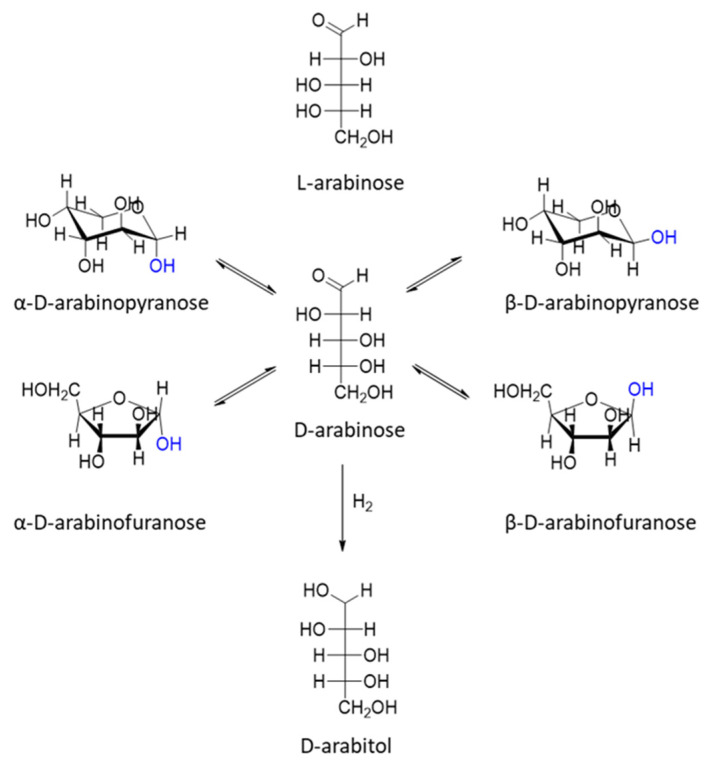
Different tautomeric and diastereomeric forms of arabinose and corresponding alcohol.

**Figure 11:**
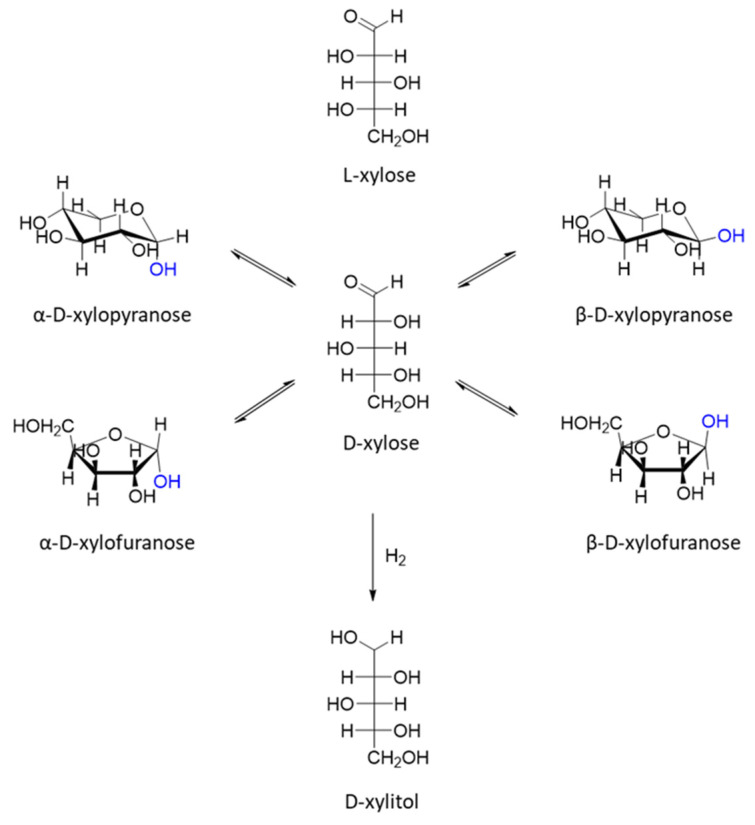
Different tautomeric and diastereomeric forms of xylose and corresponding alcohol.

The top-12 biobased molecules also include xylitol and arabinitol, which can be obtained via the hydrogenation of xylose and arabinose (Figure 10 and Figure 11). These C_5_ sugars are the products of hemicellulose hydrolysis [1,22,44].

A few examples concerning the hydrogenation of xylose and arabinose have been reported on non-noble-based catalysts. The presence of metallic Ni in the catalysts is the main requirement in achieving of sugar alcohols with good yields [32,36]. Thus, the reduction of Ni precursor to Ni^0^ and its stabilization is the main challenge in the preparation of catalysts for sugar hydrogenation. Morales et al. noted that Ni-containing mixed oxides can be used as precursors for the preparation of catalytic systems containing Ni^0^ [32]. A series of Nd_1-x_Ce_x_Al_0.162_Ni_0.838_O_3_ (x = 0.0, 0.1, 0.5, 0.7) mixed oxides was used as precursors to prepare Ni reduced catalysts for the catalytic hydrogenation of xylose to xylitol. It should be noted that Nd was used as a stabilizer of the reduced catalysts in aqueous reaction medium. The mixed oxide catalyst Nd_1_Al_0.162_Ni_0.838_O_3_ containing 20% wt. of Ni showed the highest xylose conversion of 70% at 100 °C and P (H_2_) = 25 atm after 6 h of reaction in water. Unfortunately, the xylitol selectivity reached only 53% due to side reactions including isomerization on acid sites and retro-aldol reaction. Nevertheless, the catalyst showed stable work and no Ni leaching was observed. 

The importance of the presence of Ni^0^ to achieve a high hydrogenation activity was reported by Yamaguchi et al. [35]. It was shown that the combination of nano-Ni_2_P and hydrotalcite (HT) support Mg_6_Al_2_CO_3_(OH)_16_∙4(H_2_O) played a key role in the high catalytic performance of the catalyst, due to nano-Ni_2_P activating H_2_, while HT acts as an electron donor to Ni and also can serve as activator of the xylose C-O group. According to XPS and EXAFS data, Ni in the catalyst was in Ni^0^ state and remained unchanged after the reaction. The nano-Ni_2_P/HT catalyst (0.91% wt. of Ni) allowed full xylose conversion with xylitol yield >99% after 2 h of reaction in water at 100 °C and hydrogen pressure of 20 atm. The catalyst showed stable work for at least five runs with a negligible drop in conversion, less than 6%. The authors have also shown that nano-Ni_2_P/HT catalyst was also active in disaccharide hydrogenation, i.e., maltose was effectively converted to maltitol [45].

Recently, Sadier et al. reported that supported Ni-Fe nanoalloy exhibited excellent activity and stability in xylose hydrogenation to xylitol [34]. The catalyst 26%Ni-16%Fe/SiO_2_ (Ni:Fe = 62:38 at.) prepared by deposition-precipitation method (DPU) allowed complete xylose conversion with xylitol selectivity of 98% in 4 h already at 80 °C and hydrogen pressure of 20 atm. The catalyst was shown to be stable for at least 3 runs, while monometallic 40%Ni/SiO_2_ lost its activity even after the first cycle. The deactivation of monometallic Ni catalyst was caused by the formation of layered Ni(OH)_2_ or phyllosilicate phase during the reaction, which was proved by XRD. At the same time, the formation of layered Ni(OH)_2_ or phyllosilicate was observed to be negligible in the spent Ni-Fe/SiO_2_ sample. It seems that expanded Fe-rich shell in Ni-Fe alloy blocks the total conversion of the particles to phyllosilicate seen for Ni; therefore, Ni-Fe catalysts showed stable work. 

Interestingly, Co/SiO_2_ catalyst was shown to be very active and selective in xylose hydrogenation to xylitol [33]. Xylitol conversion over the reduced 10%Co/SiO_2_ prepared by convenient incipient wetness impregnation method (IWI) reached 99% in 2 h with xylitol selectivity of 90% at 140 °C and P (H_2_) = 50 atm. However, the catalyst deactivated from run to run and conversion fell to 58% after four cycles, while the selectivity to xylitol remained 100%. The deactivation was caused by Co leaching and carbonaceous species deposition on the catalyst surface.

Continuous-flow hydrogenation of sugars is of high interest due to it being able to help to overcome the selectivity problem and make the process more suitable for industrial application [46,47,48]. Scholz et al. used hydrotalcite-derived Cu_6-x_Ni_x_Al_2_ catalysts for the transfer hydrogenation, which is very unusual for sugar hydrogenation, of a number of substrates, i.e., glucose, fructose, xylose, and arabinose, in a flow regime [36]. The best performance was achieved on Cu_3_Ni_3_Al_2_ catalyst using 1,4-butanediol as a hydrogen donor. Glucose conversion up to 90% with selectivity to sorbitol of 70% was achieved in 11 min at 150 °C in 1,4-butanedion, and γ-butyrolactone was mainly formed from 1,4-butanediol releasing two equivalents of hydrogen. Notably, g-butyrolactone is also among the 12 top-valued bio-derived molecules, which makes the overall process more appealing. Under the same conditions, xylitol and arabitol were obtained with selectivity up to 73% at xylose and arabinose conversion reached 85 and 90%, respectively. The authors account the superiority of this catalyst to the metallic phase formation and optimal interaction between copper and nickel in intermetallic compound. The prepared catalysts showed stable work in 50 h, despite a small drop in activity in the first 5 h of reaction due to adsorption of by-products and catalyst sintering. It should be added, that Molybdenum-promoted Raney nickel was also shown to be active and stable under continuous-flow hydrogenation conditions using 1,4-butanediol as a hydrogen donor [49]. Thus, glucose was completely converted to sorbitol with a selectivity of 90% (the other product was mannitol) at 130 °C and a spatial velocity of 0.04 min^−1^. Moreover, the conversion and product selectivity remained stable for at least 100 h.

## 5. Hydrolytic Cellulose Hydrogenation to Hexitols

**Figure 12:**
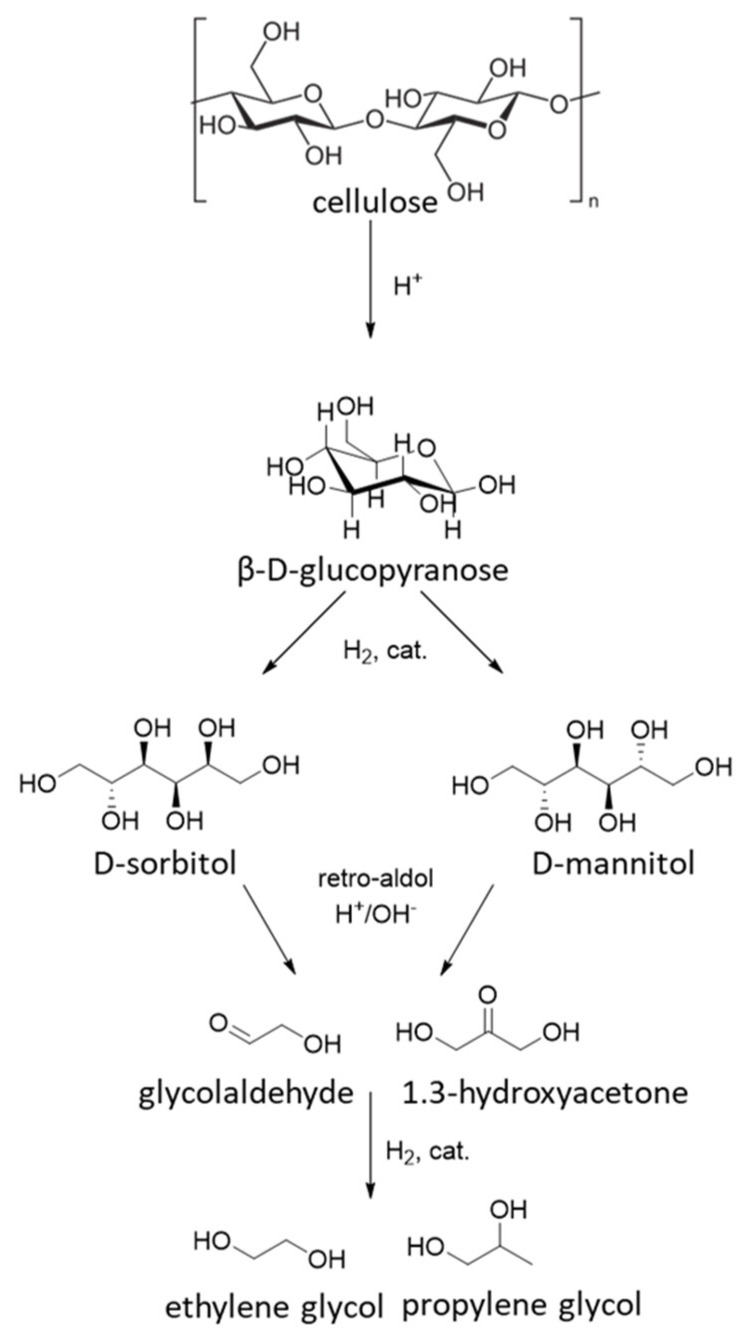
Hydrolytic hydrogenation of cellulose to hexitols and C_2_-C_3_ glycols.

Sugar alcohols such as sorbitol and mannitol can be obtained directly through one-pot process of hydrolytic hydrogenation of cellulose in water. The main challenge in such a process is a chemocatalytic cleavage of β-1,4-glycosidic bonds in the polymer chain of cellulose to form sugar monomers, which is subsequently hydrogenated to sugar alcohol. Thus, the process includes two successive steps: hydrolysis and hydrogenation. Therefore, catalytic systems should comprise both acid sites for hydrolysis and metal sites for hydrogen activation. Besides the excellent hydrogenation activity, the catalyst should have a higher activation barrier for the dehydrogenation and hydrogenolysis of hexitols than desorption energy of hexitols to favor hexitol desorption rather than their further undesired transformation [50] (Figure 12).

Many works are devoted to different combination of Ru-based catalysts and mineral acids or Ru catalysts supported on the carrier with acid sites [51,52], while data on hydrolytic hydrogenation over the catalysts with non-noble metal are rarely reported. In the literature on cellulose conversion to hexitols conducted on catalysts with base metals, Ni-containing catalysts were mostly studied [53] (Table 2). It should be noted that the main challenge in hydrolytic hydrogenation of cellulose is the selectivity because the acid sites on catalyst or acids used as co-catalyst induce the retro-aldol reaction of intermediate glucose and fructose giving rise to glycol aldehyde or 1,3-dihydroxyacetone [52]. Moreover, the formation of hexitols from cellulose require the use of much higher temperatures (>200 °C) than hydrogenation of monosaccharides because of the need to induce the rate-limiting cellulose hydrolysis step [53]. The high crystallinity of cellulose is a big issue in its processing. Therefore, different ways of cellulose treatment are used before the reaction, i.e., ball-milling, acid treatment, or the use of microcrystalline cellulose (MCC).

Ni NPs supported on zeolites seem to be the most evident catalytic system that combine both hydrogenation activity and acid function. Nickel multiply-twinned particles (Ni MTPs) imbedded onto ZSM-5 provided cellulose conversion as high as 94% with hexitols selectivity of 67% after 2.5 h of reaction at 240 °C and a hydrogen pressure of 40 atm [54]. The authors suggested that Ni NPs with a mean particle size of 18 nm and multiple twinned structure with exposed Ni (1 1 1) plain and Ni^δ+^ species are the active sites for sugar adsorption and hydrogen activation. In contrast to ZSM-5 with strong acid sites, Ni MTPs/ZSM-5 catalyst possessed acidic sites of medium strength, which result in supressed dehydrogenation activity of the catalyst and good yield of sorbitol and mannitol [54,56]. However, the catalyst was not stable. Hydrogenation of cellobiose, a glucose dimer connected by 1,4-β-glycosidic bond, over the catalyst 40%Ni MTPs/ZSM-5 of the same structure proceeded to complete substrate conversion and the selectivity to sorbitol and mannitol reached 82% after 6 h of reaction at 240 °C and hydrogen pressure of 40 atm [50]. It was shown that the use Al_2_O_3_ and SiO_2_ instead of ZSM-5 resulted in a lower selectivity to hexitols. Acid-base pairs on the surface of Al_2_O_3_ and SiO_2_ resulted in higher dehydrogenation activity of Ni supported catalysts and caused undesirable retro-aldol reaction. 

Zhang et al. used cellulose not only as a substrate in hydrolytic hydrogenation reaction but also for the synthesis of mesoporous ZSM-5 zeolite, which was used as a support for a Ni catalyst [55]. Ni supported on nano crystalline cellulose templated ZSM-5 (NCC-ZSM-5) with a surface area of 430–480 m^2^/g and mesoporous structure with pore volume = 0.8–0.9 cm^3^/g was able to convert microcrystalline cellulose (MCC) to sorbitol and mannitol with a selectivity of 60% and 87% of cellulose conversion after 2.5 h of reaction under hydrogen pressure as high as 40 atm and a temperature of 240 °C. The presence of strong acid sites, high specific surface area, optimal mesopore structure with a high accessibility of acid sites and favourable Ni NPs size (25–27 nm) were responsible for the high performance of Ni/NCC-ZSM-5 catalyst. Catalyst deactivation was observed both at high and low conversions, which was attributed to Ni particle agglomeration and damage of the ZSM-5 mesopore structure.

Ni nanoparticles supported on carbonaceous materials represent an alternative catalytic system for cellulose hydrolytic hydrogenation. It was shown that such a convenient catalyst as Ni supported on carbon black or mesoporous carbon was extremely effective in the hydrolytic hydrogenation of cellulose to sorbitol and mannitol [59,60]. The catalyst 50%Ni/C afforded cellulose conversion of 90% with selectivity to hexitols of 71% already at 210 °C and P(H_2_) = 50 atm after 6 h of reaction. Interestingly, the higher the Ni content, the better the catalyst stability, because of the growth of Ni nanoparticles. After 2 h of reaction, the Ni NPs size in 50%Ni/C increased from 11 to 28 nm and remained unchanged until the reaction was finished. However, the catalyst gradually lost activity because of Ni oxidation with the formation of the outermost layer of Ni(OH)_2_. 

An unusual strategy in the development of a catalytic system for cellulose hydrolytic hydrogenation was proposed by Van de Vyver et al. [57,58]. The authors noted the very limited accessibility of the active catalytic sites, especially for porous catalysts, for cellulose molecule. The authors used the concept of high accessibility of Ni NPs supported on the tips of carbon nanofibers (CNF), which entangled around the water-insoluble cellulose matrix. The catalytic system Ni/CNF/Al_2_O_3_ was prepared by catalytic vapour deposition (CVD) of methane onto Ni/Al_2_O_3_ sample with subsequent calcination and reduction at 500 °C. This method led to the formation of rather big Ni NPs with a size almost equal to a carbon fibre diameter of 60–100 nm, even for the sample with 3 % wt. of Ni. The conversion of ball-milled cellulose (at 190 °C for 24 h) over 3%Ni/CNF/Al_2_O_3_ was 92% with selectivity to sorbitol and mannitol 61% after 4 h at 230 °C and H_2_ pressure of 60 atm. The temperature of the reaction could be significantly lowered to 190 °C. However, 88% cellulose conversion with 63% selectivity to hexitols was obtained only after 24 h of reaction. The authors concluded that cellulose hydrolysis can be catalysed by both H^+^ ions reversibly formed in situ in hot water [64] and Brønsted acid sites on the Al_2_O_3_ support. The 3%Ni/CNF/Al_2_O_3_ catalyst showed good stability and could be reused for at least three experiments. To enhance the acidity of the Ni/CNF catalysts and better anchor Ni NPs on carbon nanofibers, CNF grown on Ni/Al_2_O_3_ was treated in HNO_3_ at 110 °C for 1 h [58]. The acid-treated CNF was used as the support for Ni NPs deposited by impregnation technique. Thus, acid-treated Ni/CNF with a Ni content of 7.5% wt. afforded cellulose conversion of 93% with an extremely high selectivity to hexitols reaching 82% at 190 °C and H_2_ pressure of 60 atm in 24 h of reaction. The authors noted that high activity of 7.5%Ni/CNF was attributed to the optimal balance between exposed Ni surface atoms (n Ni) and acid sites (n A), which should be 0.7 < n A/n Ni < 1.1. At lower n A/n Ni ratio, the sugar alcohols become more susceptible for C-C bond hydrogenolysis, while at higher n A/n Ni values it is more likely that the glucose formed is subjected to side acid-catalyzed degradation (retro-aldol reaction) rather than being hydrogenated to sorbitol and mannitol.

Metal phosphides were also reported to be active in cellulose hydrolytic hydrogenation. Nickel phosphide phase Ni_2_P supported on active carbon possessed the required bifunctionality combining acid and hydrogenation sites [61,62]. 16%Ni_2_P/AC afforded complete cellulose conversion with the selectivity to sorbitol and mannitol 48% and 5%, respectively, at 225 °C under a hydrogen pressure of 60 atm after 1.5 h of the reaction [61]. The authors showed that the catalyst activity and selectivity significantly depended on the Ni/P ratio, i.e., the ratio of exposed hydrogenation sites and acid catalytic centres. However, the catalyst was unstable under hydrothermal conditions, and significant phosphorus leaching was observed. The formation of Ni phosphide phase (Ni_2_P) was essential for direct cellulose conversion to sorbitol with a high yield. Yang et al. demonstrated that during the reaction, crystalline Ni_2_P phase transformed to a highly dispersed amorphous Ni phosphide (ANP) [62]. Thus, a high activity of the 10%Ni-3.2%P/AC catalyst was attributed to ANP formation. Cellulose hydrolytic hydrogenation over 10%Ni-3.2%P/AC catalyst proceeded with conversion of 90% and selectivity to sorbitol as high as 68% at 230 °C and P (H_2_) = 40 atm already in 0.7 h of reaction. The catalyst had a low reusability because of phosphorus leached in form of H_3_PO_4_, which in turn caused catalyst structure transformation from ANP to Ni crystallites of approximately 30 nm. Liu et.at. used zirconium phosphate Zr(HPO_4_)_2_·H_2_O (ZrP) as a support for Ni NPs in hydrolytic cellulose hydrogenation [63]. The activity and selectivity of the catalysts was tuned by adjusting the P/Zr ratio. For Ni/ZrP*_x_* with P/Zr ≥ 2 a higher total acid content was determined than that with P/Zr ≤ 1.5. The best performance was observed for Ni supported on Zr phosphate with P/Zr ratio of 2 (ZrP_2_). For the 20%/ZrP_2_ catalyst, the sorbitol yield reached 60% at 200 °C and H_2_ pressure of 50 atm in 5 h. Moreover, this catalyst was rather stable. Only a slight decrease in sorbitol yield (ca. 3%) was observed after three experiments, which accounted for negligible Ni leaching during the reaction. 

**Figure 13:**
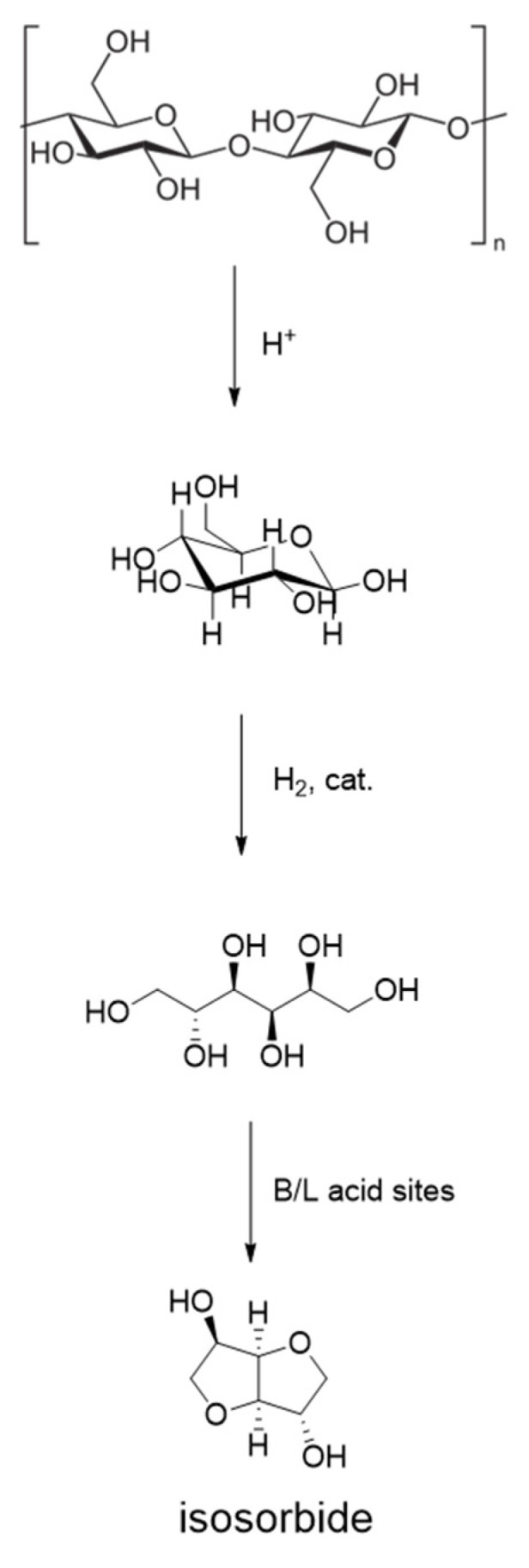
Direct conversion of cellulose to isosorbide.

Interestingly, Ni supported on niobium phosphate afforded direct one-pot conversion of cellulose to value-added isosorbide [65]. This is a first example on non-noble catalyst active in complex three-step cellulose conversion to isosorbide; other studies have been devoted to Ru/C catalysts combined with acids as co-catalysts or Ru supported on solid acids [66]. The catalytic processing of cellulose to sorbitol includes hydrolysis, hydrogenation and dehydration reactions (Figure 13). The presence of both Lewis (L) and Brønsted (B) acid sites together with hydrogenation sites enabled 70%Ni/NbOPO_4_ bifunctional catalyst provide full cellulose conversion with isosorbide yield of 47% at 200 °C and H_2_ pressure of 30 atm in water after 24 h of reaction. More importantly, the catalyst was stable for at least five experiments without any loss of activity [65]. 

## 6. Conclusions

The green route in biomass conversion is still a new-born field with many challenges. The replacement of existing catalysts based on noble metals (Ru, Pd, Pt) with non-noble ones is one of the fundamental problems that restricts the implementation of new green strategies in chemical technology. We hope that this review can help in analysing the current state of art in the hydrogenation of bio-available molecules such as simple sugar and readily available cellulosic feedstock in the production of value-added sugar alcohols and their derivatives in the presence of non-noble metal catalysts.

Ni and Cu-containing mono-and bimetallic catalysts represent a prominent alternative to catalytic systems based on noble metals in the hydrogenation of sugars. Moreover, direct hydrolytic hydrogenation of cellulose to sugar alcohols such as sorbitol and mannitol or even value-added isosorbide can be performed on non-noble Ni-containing catalysts. However, the main challenge in obtaining active and selective catalysts for the hydrogenation of both monosaccharides and cellulose to sugar alcohols is the formation and stabilisation of the active components in metallic form. As was shown, Ni^0^ and Cu^0^ are believed to be the active sites for hydrogen activation and sugar adsorption predominantly in cyclic form. Stabilisation of the active components in metallic form can be achieved by the formation of bimetallic nanoalloys or specific phases such as phosphides or hydrotalcite derived mixed oxides (with their subsequent reduction). The choice of a support for well-dispersed nanoparticles also plays an important role in catalytic hydrogenation. Besides the stabilisation function for nanoparticles, the support can also provide the active sites for the substrate adsorption and additional acid/base centres, which can tune the selectivity of the process. In most cases, even if the catalysts are highly active and selective in hydrogenation, they are not stable under hydrothermal reaction conditions as the reaction is performed in water. Leaching of the active component as well as its oxidation or inactive hydroxide formation (for instance, Ni(OH)_2_) causing gradual loss in the catalyst activity are the main problems. Thus, the reusability problem has not yet been completely solved.

The development of new strategies and new types of catalytic systems for sugar hydrogenation and cellulose hydrolytic hydrogenation are of great interest and represent a large field for further investigation, especially in view of sustainable and eco-friendly catalytic processes. For example, zeolite-supported Ni catalysts showed good performance for cellulose transformation to sorbitol. However, the catalyst was not stable because of Ni leaching and the agglomeration of Ni nanoparticles during the reaction. The use of Ni-MOF catalysts can be an effective way in both Ni stabilization as well as providing optimal balance between the hydrogenation and acid sites to achieve high selectivity in hexitol formation. Performing the hydrogenation reactions in a flow regime can also be important, not only in view of making the process more convenient for large-scale realization, but also in enhancing the selectivity in the obtainment of sugar alcohols, inhibiting consecutive side reactions. Rapid heating under microwave irradiation can significantly reduce the reaction time, which can also lead to an increase in the selectivity of sugar alcohol formation and reduce the influence of the reaction conditions on the catalyst structures, making them more durable.

## Figures and Tables

**Table 1:** Hydrogenation of C_5_-C_6_ sugars over the catalysts with non-noble metals.

Catalyst	Regime	Substrate	Product	t, °C	P (H_2_), Atm	Time, h	Solvent	Sub/Met	X, %	S, %	Refs.
Ni-Co nanoalloy (1:1)	Batch	D-glucose	D-sorbitol	90	30	4	H_2_O	0.14 mol/g	97	99	[23]
Ni-B	Batch	D-glucose	D-sorbitol	120	40	2	H_2_O	0.3 mol/g	85	n.d.	[24]
Ni/NiO Mesopore Catalyst	Batch	D-glucose	D-sorbitol	130	50	n.d.	H_2_O	0.13 mol/g	95	88	[25]
5%Ni/Al_2_O_3_ (Ni^0^)	Batch	D-glucose	D-sorbitol	130	30	4	H_2_O	19.6 mol/mol (0.34 mol/g Ni)	60	80	[26]
5%Cu/SiO_2_ (Cu^0^)	Batch	D-glucose	D-sorbitol	130	30	4	H_2_O	19.6 mol/mol	45	89	[22]
Ni_1.85_Cu_1_Al_1.15_	Batch	D-glucose	D-sorbitol	120	30	3	H_2_O	0.03 mol/g	80	87	[27]
8%Fe-8%Ni/CB	Batch	D-glucose	D-sorbitol	140	30	6	H_2_O	16.5 mol/mol	71	70	[28]
80%Cu/SiO_2_	Batch	D-fructose	D-mannitol	120	35	10	1-butanol	2.2 mol/mol	>99	83	[29]
Ni_4.63_Cu_1_Al_1.82_Fe_0.79_	Batch	D-fructose	D-mannitol	110	30	2	H_2_O	0.03 mol/g	>99	57	[30]
5%Cu-7%Ni/SiO_2_	Batch	D-fructose	D-mannitol	100	40	2	H_2_O	8.3 mol/mol	>99	73	[31]
			D-sorbitol							24	
11%Cu/SiO_2_	Batch	D-fructose	D-mannitol	100	40	6	H_2_O	9.5 mol/mol	95	75	[29]
			D-sorbitol							22	
9%Ni/SiO_2_	Batch	D-fructose	D-mannitol	100	40	6	H_2_O	10.5 mol/mol	55	55	[29]
Nd_1_Al_0.162_Ni_0.838_O_3_ (20% wt Ni)	Batch	D-xylose	D-xylitol	100	25	6	H_2_O	9.8 mol/mol	70	53	[32]
10%Co/SiO_2_	Batch	D-xylose	D-xylitol	140	50	2	H_2_O	78.5 mol/mol	>99	90	[33]
26%Ni-16%Fe/SiO_2_	Batch	D-xylose	D-xylitol	80	20	4	H_2_O	10.6 mol/mol	>99	98	[34]
Ni_2_P/HT (0.91% wt. of Ni)	Batch	D-xylose	D-xylitol	100	20	2	H_2_O	16 mol/mol	>99	>99	[35]
Cu_3_Ni_3_Al_2_	Flow	glucose	sorbitol	150	hydrogen donor 1,4-butanediol	0.19	H_2_O	6-45 mol/g·min	87	70	[36]
		fructose	mannitol/sorbitol						85	70/19	
		xylose	xylitol						85	71	
		arabinose	arabinitol						90	73	

**Table 2:** Hydrolytic hydrogenation of cellulose over the catalysts with nonnoble metals.

Catalyst	Substrate	Product	T, ^°^C	P (H_2_), Atm	Time, h	Solvent	sub/cat	X, %	S, %	Refs.
Ni MTPs/ZSM-5	Cellulose	Sorbitol + Mannitol	240	40	2.5	H_2_O	0.3 g/100 mg	94	67	[54]
Ni/ZSM-5 (Ni 40 % wt.)	Cellobiose	Sorbitol + Mannitol	240	40	4	H_2_O	0.2 g/100 mg	>99	82	[50]
Ni/Al_2_O_3_								>99	29	
Ni/SiO_2_								>99	44	
Ni/NCC-ZSM-5 nanocrystalline cellulose-templated mesoporous ZSM-5	Microcrystalline Cellulose (MCC)	Sorbitol + Mannitol	240	40	2.5	H_2_O	0.1 g/50 mg	87	68	[55]
17%Ni/ZSM-5	Microcrystalline Cellulose (MCC)	Sorbitol + Mannitol	240	40	2.5	H_2_O	0.2 g/100 mg	87	67	[56]
40%Ni/ZSM-5								86	57	
40%Ni/Al_2_O_3_								85	16	
40%Ni/SiO_2_								84	19	
3%Ni/CNF/Al_2_O_3_	Ball-Milled Cellulose at 190 °C for 24 h	Sorbitol + Mannitol	230	60	4	H_2_O	1 g/500 mg	92	61	[57]
3%Ni/CNF/Al_2_O_3_			190	60	24			88	63	
7.5%Ni/CNF HNO_3_ treated (Ni surface atom 26.9 μmol/g cat)	Ball-Milled Cellulose at 190 °C for 24 h	Sorbitol + Mannitol	190	60	24	H_2_O	1 g/500 mg	93	82	[58]
2.6%Ni/CNF HNO_3_ treated (Ni surface atom 8.1 μmol/g cat)								91	54	
1%Ir-5%Ni/MC	Microcrystalline Cellulose (MCC)	Sorbitol + Mannitol	245	60	0.5	H_2_O	0.5 g/150 mg	>99	58	[59]
1%Rh-5%Ni/MC								>99	60	
20%Ni/MC								85	51	
20%Ni/AC								62	32	
50%Ni/C	Ball-Milled Cellulose	Sorbitol + Mannitol	210	50	6	H_2_O	0.3 g/50 mg	90	71	[60]
16%Ni_2_P/AC	Cellulose	Sorbitol	225	60	1.5	H_2_O	0.5 g/150 mg	>99	48	[61]
10%Ni-3.2%P/AC (Ni_2_P)	Ball-Milled Cellulose	Sorbitol	230	40	0.7	H_2_O	0.162 g/50 mg	90	68	[62]
20%Ni/ZrP_2_	Cellulose	Sorbitol	200	50	5	H_2_O	0.05 g/50 mg		61% yield	[63]

## Data Availability

All the reported data were taken from the published materials referenced below.
